# Differences in Breastfeeding Duration by Maternal HIV Status: A Pooled Analysis of Nationally Representative Surveys in Sub-Saharan Africa

**DOI:** 10.1097/QAI.0000000000003317

**Published:** 2024-01-04

**Authors:** Robert Glaubius, John Stover, Leigh F. Johnson, Severin G. Mahiane, Mary I. Mahy, Jeffrey W. Eaton

**Affiliations:** a Center for Modeling, Planning and Policy Analysis, Avenir Health, Glastonbury, CT;; b Centre for Infectious Disease Epidemiology and Research, University of Cape Town, Cape Town, South Africa;; c Data for Impact, UNAIDS, Geneva, Switzerland;; d Center for Communicable Disease Dynamics, Department of Epidemiology, Harvard T. H. Chan School of Public Health, Boston, MA; and; e MRC Centre for Global Infectious Disease Analysis, School of Public Health, Imperial College London, London, United Kingdom.

**Keywords:** HIV/AIDS, breastfeeding, vertical transmission, sub-Saharan Africa

## Abstract

**Background:**

Breastfeeding improves child survival but is a source of mother-to-child HIV transmission among women with unsuppressed HIV infection. Estimated HIV incidence in children is sensitive to breastfeeding duration among mothers living with HIV (MLHIV). Breastfeeding duration may vary according to maternal HIV status.

**Setting:**

Sub-Saharan Africa.

**Methods:**

We analyzed pooled data from nationally representative household surveys conducted during 2003–2019 that included HIV testing and elicited breastfeeding practices. We fitted survival models of breastfeeding duration by country, year, and maternal HIV status for 4 sub-Saharan African regions (Eastern, Central, Southern, and Western).

**Results:**

Data were obtained from 65 surveys in 31 countries. In 2010, breastfeeding in the first month of life (“initial breastfeeding”) among MLHIV ranged from 69.1% (95% credible interval: 68–79.9) in Southern Africa to 93.4% (92.7–98.0) in Western Africa. Median breastfeeding duration among MLHIV was the shortest in Southern Africa at 15.6 (14.2–16.3) months and the longest in Eastern Africa at 22.0 (21.7–22.5) months. By comparison, HIV-negative mothers were more likely to breastfeed initially (91.0%–98.7% across regions) and for longer duration (median 18.3–24.6 months across regions). Initial breastfeeding and median breastfeeding duration decreased during 2005–2015 in most regions and did not increase in any region regardless of maternal HIV status.

**Conclusions:**

MLHIV in sub-Saharan Africa are less likely to breastfeed initially and stop breastfeeding sooner than HIV-negative mothers. Since 2020, UNAIDS-supported HIV estimates have accounted for this shorter breastfeeding exposure among HIV-exposed children. MLHIV need support to enable optimal breastfeeding practices and to adhere to antiretroviral therapy for HIV treatment and prevention of postnatal mother-to-child transmission.

## Abstract 2

Supplemental Digital Content is Available in the Text.

## INTRODUCTION

UNAIDS estimated that 130,000 (90,000–210,000) children acquired HIV in 2022.^1^ Of these, 82% occurred in sub-Saharan Africa, and 42% of new child infections were estimated to occur during breastfeeding.^2^ These estimates were derived through a multistep modeling process in which infants born to mothers living with HIV (MLHIV) are exposed to mother-to-child HIV transmission at the time of delivery and a monthly probability thereafter in each month of breastfeeding.^3,4^ Therefore, estimates of new child HIV infections are sensitive to inputs about the duration of breastfeeding among MLHIV, along with other determinants such as stage of maternal HIV infection and coverage of antiretrovirals for prevention of mother-to-child transmission (PMTCT). Before the 2020 round of UNAIDS HIV estimates, new HIV infections in children in sub-Saharan Africa were often estimated assuming breastfeeding durations measured in nationally representative household surveys among all mothers regardless of maternal HIV status.^3^

Breastfeeding reduces childhood morbidity and mortality,^5^ but breastfeeding practices may differ by maternal HIV status. Numerous individual and sociocultural factors affect women's breastfeeding decisions.^6^ MLHIV face additional challenges, including potential of transmitting HIV, HIV-related stigma, and uncertainty in the face of changing guidance about breastfeeding recommendations for MLHIV.^6–9^ Since 2001, the World Health Organization (WHO) has recommended that mothers exclusively breastfeed infants for the first 6 months of life and continue breastfeeding with appropriate complementary foods for 24 months or more.^10,11^ Although guidance for the general population has been consistent, recommendations for MLHIV have changed with time,^9,12,13^ reflecting the accumulation of evidence for the risk of mother-to-child transmission (MTCT) of HIV,^14^ the benefit of exclusive breastfeeding for HIV-free survival during the first 6 months of life,^12,15,16^ and the effectiveness of antiretroviral drugs for PMTCT during pregnancy and breastfeeding.^13^ As of 2016, WHO guidance recommends breastfeeding for MLHIV while being supported to adhere to antiretroviral treatment, closely aligned with general population guidance.^13^ Since 2015, WHO have recommended immediate lifelong ART initiation for all HIV-positive pregnant women, which considerably reduces the risk of mother-to-child HIV transmission if women are retained on treatment and virally suppressed through the peripartum and breastfeeding periods.^17^

Many countries and international organizations, including UNAIDS, use the Spectrum software suite for HIV program planning and to estimate key HIV indicators, including new HIV infections in children.^18,19^ Estimates of postnatal new child HIV infections in Spectrum depend on breastfeeding duration among MLHIV and the risks of MTCT during breastfeeding.^4^ Country-specific estimates of breastfeeding duration among MLHIV suffer from small sample sizes. We analyzed data from nationally representative household surveys,^20,21^ with HIV testing to estimate breastfeeding duration by maternal HIV status in sub-Saharan Africa while accounting for differences in breastfeeding practices over time and between countries.

## METHODS

### Data Sources

We conducted a retrospective pooled analysis of national cross-sectional household surveys of women aged 15–49 years from sub-Saharan Africa. Surveys were included if HIV testing was performed, HIV serostatus could be linked to individual survey responses about current breastfeeding practices at the time of survey (e.g., “Are you still breastfeeding [CHILD NAME]?”), and data on births in the past 3 or more years were collected. We excluded surveys that asked only about retrospective breastfeeding duration (“For how many months did you breastfeed [CHILD NAME]?”) but not about current breastfeeding practice because of potential biases when using recalled durations to elicit current breastfeeding practices at the time of the survey. We estimated current breastfeeding practices among mothers who gave birth in the 36 months preceding the survey. Mothers with an indeterminant HIV test result or missing breastfeeding response were excluded.

We obtained survey microdata from Demographic and Health Surveys (DHS) and AIDS Indicator Surveys (AIS) through the DHS Program,^22^ except for 2007 and 2012 AIS data of Kenya, which are available from National Bureau of Statistics website of Kenya.^23,24^ Data from Population-Based HIV Impact Assessment (PHIA) surveys were obtained from the PHIA project.^25^ Multiple Indicator Cluster Surveys do not include HIV testing in most countries and were excluded. All included surveys used a standard stratified 2-stage cluster sampling design. Observations were weighted to account for different sampling probabilities of survey participation. We used normalized “HIV weights” for analysis of DHS and AIS data because all analyses of breastfeeding practices by HIV status were restricted to women who participated in HIV serological testing.

### Breastfeeding Model

We specified a model for breastfeeding duration consisting of a proportion *u* of mothers who breastfed initially (ie, in the first month after giving birth), after which their breastfeeding duration followed a log-logistic distribution parametric survival model with median *m* and shape *s*. Parameters for both the initial proportion breastfeeding and the median and shape parameters of the log-logistic distribution were specified as linear models for each region (Eastern, Central, Southern, or Western) of sub-Saharan Africa. We used DHS Program definitions of these regions.^26^

We let b(k,t,a,h) be the proportion of mothers who were currently breastfeeding in country *k* and year *t*, stratified by the child's age in months *a* (0–1, 2–3, … 34–35 months) and maternal HIV status *h*,

(1) b(k,t,a,h)=u(k,t,h)⋅F(a;m(k,t,h),s(k,t))

The term u(k,t,h) represents the proportion of mothers who breastfed, and F(a;m,s) is the log-logistic survival function with median *m* and shape *s*,

(2) $$F\left(a;m,s\right)=1-{\left[1+{\left(a/m\right)}^{-s}\right]}^{-1}$$

The proportion of mothers who breastfed initially and their median breastfeeding duration varied by country, year, and maternal HIV status, whereas the shape parameter varied by country and year:

(3) $$\text{logit}\;u\left(k,t,h\right)=\text{logit}\;\theta_{k}+\theta_{r}^{\prime }\cdot t+\left\{\rho_{r}+\rho_{r}^{\prime }\cdot t\right\}$$

(4) $$\ln m\left(k,t,h\right)=\mu_{k}+\mu_{r}^{\prime }\cdot t+\left\{\lambda_{r}+\lambda_{r}^{\prime }\cdot t\right\}$$

(5) $$\ln s\left(k,t\right)=\sigma_{k}+\sigma_{r}^{\prime }\cdot t$$

Terms in braces above (“{}”) applied to MLHIV only, all other terms applied to any mother regardless of HIV status. Parameters $\theta_{k}$, $\mu_{k}$, and $\sigma_{k}$ were estimated for each country, whereas changes in breastfeeding over time ($\theta_{r}^{\prime }$, $\mu_{r}^{\prime }$, $\sigma_{r}^{\prime }$), by HIV status ($\rho_{r},\lambda_{r}$) and interactions between HIV and time ($\rho_{r}^{\prime }$, $\lambda_{r}^{\prime }$) were estimated for each region r because there were generally too few surveys per country and too few MLHIV per survey to estimate HIV or temporal effects at the country level robustly.

We assigned surveys to the year closest to the midpoint of the data collection interval. We standardized survey years so that t=0 corresponds to reference year 2010 near the midpoint of available surveys so that country-level parameter estimates can be interpreted as reference year values.

We report *initial breastfeeding* as the proportion of mothers who breastfed in the first month after giving birth. We report *median breastfeeding duration* as the conditional median duration among mothers who breastfed initially. Note that this conditional median is longer than the (unconditional) median duration among all mothers except when 100% of mothers breastfed initially.

We estimate breastfeeding durations throughout the period spanned by surveys in each region of sub-Saharan Africa. We focus quantitative comparisons on years 2005, 2010, and 2015 because most surveys with HIV testing were conducted in this period.

### Parameter Estimation

We estimated model parameters through Bayesian methods using Stan^27^ in R 4.2.2.^28^ We fitted models to aggregate counts of mothers from each survey, stratified by country, survey year, maternal HIV status, months since last birth, and current breastfeeding status. We assumed noninformative flat prior distributions on all model parameters. We derived the likelihood by assuming the number of currently breastfeeding mothers was binomially distributed given the number of mothers in each stratum. We used the No U-Turn Sampler (NUTS) in Stan to obtain 4000 samples from the posterior distribution. Point estimates were obtained from the parameter sample with highest posterior density; we calculated 95% credible intervals (CrIs) as the central 95% quantile range across samples.

R code for this analysis is available at https://github.com/rlglaubius/InfantFeedingAnalysis. This code is restricted to use DHS and AIS microdata, which can be accessed using the rdhs package in R.^29^ Note that this excludes data from PHIA and AIS surveys of Kenya, which are not part of the DHS Program.

### Sensitivity Analyses

There were relatively few household surveys with HIV testing available for Southern Africa. We evaluated the sensitivity of breastfeeding duration estimates in the region to inclusion or exclusion of South Africa's 2016 DHS to examine the potential for misidentification of between-country differences as temporal effects.

### Effect on Pediatric HIV Estimates

We used Spectrum to estimate incident and prevalent HIV infections in children when breastfeeding duration inputs were based on mothers living with HIV or on all mothers regardless of HIV status. Detailed methods are provided in Text 1, Supplemental Digital Content, http://links.lww.com/QAI/C133.

### Ethics Statement

All analyses were secondary analyses performed on deidentified data. The survey protocols for the DHS and AIS were approved by the Internal Review Board of ICF International and by the relevant national authorities in each country.^30^ PHIA surveys were reviewed and approved by in-country ethics and regulatory bodies and the Institutional Review Boards of Columbia University Medical Center, Westat, and the CDC.

## RESULTS

**FIGURE 1.**
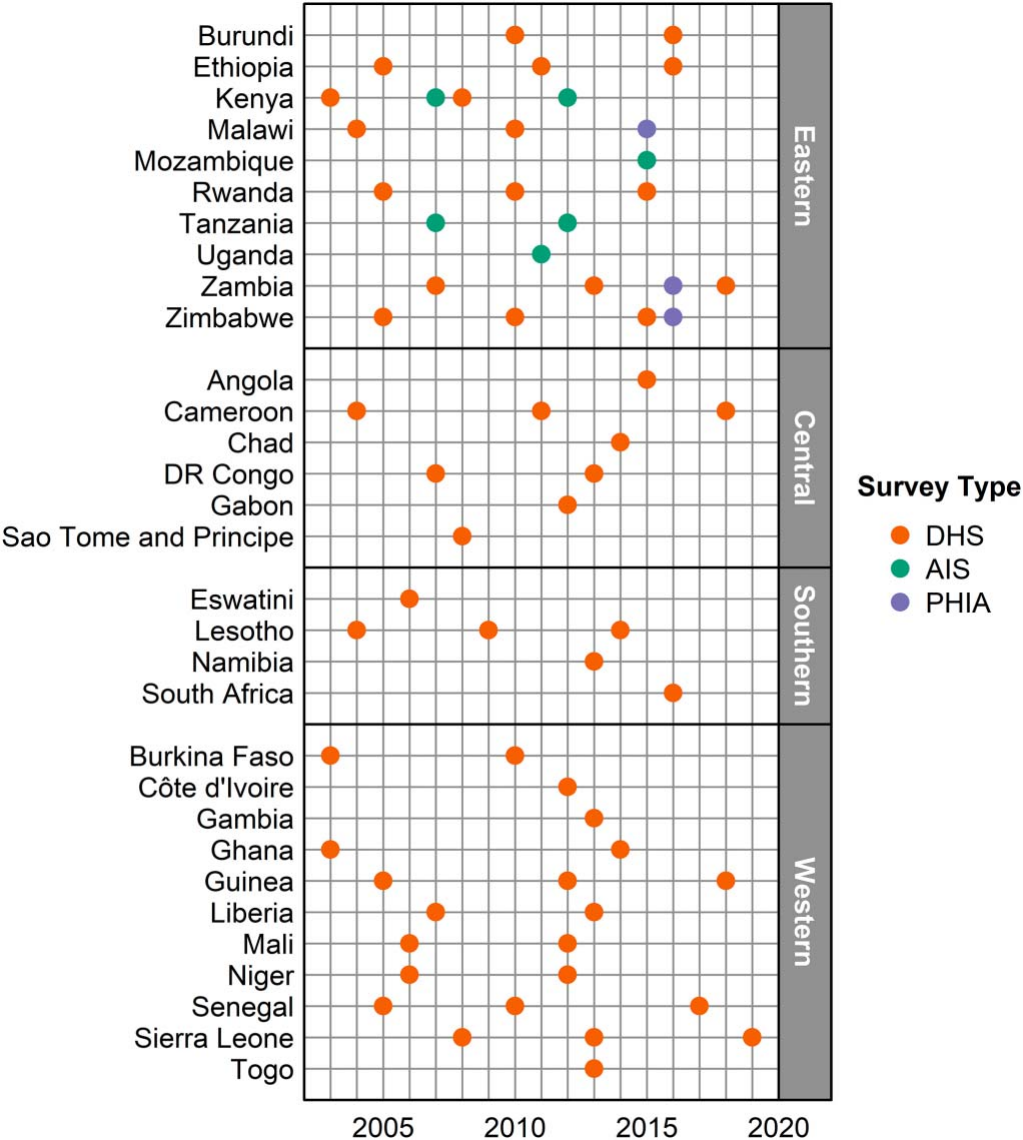
Household surveys included in analyses of breastfeeding duration. The year nearest to the midpoint is shown for surveys with fieldwork in multiple years. Two surveys (2015–2016 DHS, 2015–2016 MPHIA) were included for Malawi in 2015.

We analyzed data from 65 nationally representative household surveys (56 DHS, 6 AIS, and 3 PHIA) from 31 countries (Fig. 1). The earliest survey was in 2003 (Kenya 2003 DHS) and the most recent in 2019 (Sierra Leone 2019 DHS). Only 3 PHIAs (Malawi 2015-16, Zimbabwe 2015-16, and Zambia 2016) were included because later survey questionnaires asked about retrospective breastfeeding duration but not current breastfeeding status. Our analytic data set included 167,749 mothers, of whom 9450 were HIV positive (Table 1).

**TABLE 1.** Characteristics of Survey Respondents Included in Breastfeeding Analyses

Characteristic	HIV-Negative, N (%)	HIV-Positive, N (%)	Total, N (%)
Total	158,299 (100)	9450 (100)	167,749 (100)
Region			
Central Africa	22,510 (14)	561 (6)	23,071 (14)
Eastern Africa	81,960 (52)	6701 (71)	88,661 (53)
Southern Africa	4775 (3)	1549 (16)	6324 (4)
Western Africa	49,054 (31)	639 (7)	49,693 (30)
Period			
2003–2008	40,820 (26)	2622 (28)	43,442 (26)
2009–2014	72,354 (46)	3461 (37)	75,815 (45)
2015–2019	45,125 (29)	3367 (36)	48,492 (29)
Survey type			
AIS	19,179 (12)	1301 (14)	20,480 (12)
DHS	130,427 (82)	6931 (73)	137,358 (82)
PHIA	8693 (5)	1218 (13)	9911 (6)

Percentage values are relative to totals by HIV status and may not sum to 100% because of rounding.

### Country Estimates

**FIGURE 2.**
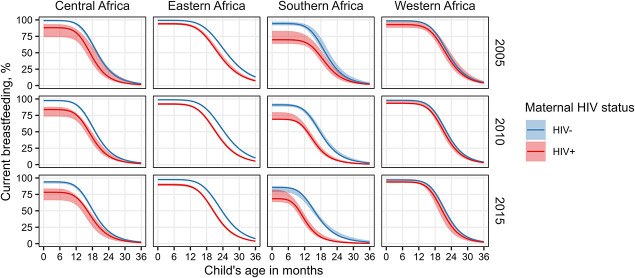
Modeled breastfeeding patterns by region and HIV status in 2005, 2010, and 2015. Regional trends are based on averages of country parameter estimates. Point estimates (solid curves) and 95% credible intervals (shaded areas) are shown.

Figure 2 shows regional average breastfeeding pattern estimates by maternal HIV status at the reference year of 2010 and time points 5 years before (2005) and after (2015). Regional breastfeeding estimates were based on regional parameter estimates (Table 1, Supplemental Digital Content, http://links.lww.com/QAI/C133) and the averages of country-specific parameter estimates (Table 2, Supplemental Digital Content, http://links.lww.com/QAI/C133). Country-specific parameters specify 2010 values for the percentage of HIV-negative mothers who breastfeed initially, the median duration of breastfeeding among HIV-negative mothers who breastfed initially, and the log-logistic distribution shape parameter (Table 2, Supplemental Digital Content, http://links.lww.com/QAI/C133). Initial breastfeeding among HIV-negative mothers was the lowest in South Africa at 81.9% (95% CrI: 74.4–90.2) and above 99% in 6 countries: Burundi, Democratic Republic of Congo, Ghana, Rwanda, São Tomé and Príncipe, and Tanzania. The median breastfeeding duration among HIV-negative mothers who breastfed initially was the shortest in Gabon (12.3 months, 95% CrI: 11.8–13.5) and at least 24 months in 9 countries (Burkina Faso, Burundi, Chad, Democratic Republic of Congo, Ethiopia, Guinea, Malawi, Rwanda, and Togo).

### Differences in Breastfeeding Duration for MLHIV

**FIGURE 3.**
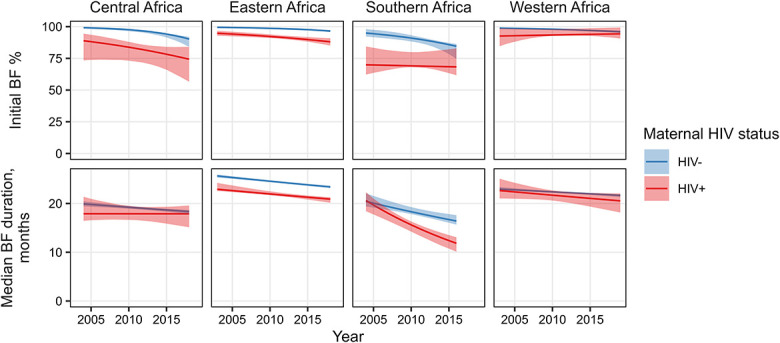
Regional estimates of initial breastfeeding proportion (top) and median breastfeeding duration (bottom). Regional estimates are based on averages of country parameter estimates. Point estimates (lines) and 95% credible intervals (shaded areas) are shown for the period between the earliest and latest surveys in each region. BF, breastfeeding.

The initial breastfeeding proportion was lower among MLHIV than HIV-negative mothers in all regions (Fig. 3, Table 3, Supplemental Digital Content, http://links.lww.com/QAI/C133). In the reference year 2010, the gap was the largest and overall breastfeeding was the lowest in Southern Africa where 69.1% (95% CrI: 68.0–79.9) MLHIV breastfed versus 91% (95% CrI: 88.3–93.2) of HIV-negative mothers. In Central Africa, the gap was also large at 83.8% (95% CrI: 73.2–88.0) of MLHIV breastfeeding versus 97.6% (95% CrI: 96.4–98.0) of HIV-negative mothers. In Eastern Africa, 92.2% (95% CrI: 91.0–93.9) of MLHIV and 98.7% (95% CrI: 98.5–98.9) of HIV-negative mothers breastfed, and in Western Africa, 93.4% (95% CrI: 92.7–98.0) of MLHIV and 98.0% (95% CrI: 97.9–98.4) of HIV-negative mothers initially breastfed. Initial breastfeeding decreased over time among HIV-negative women in all regions, and among MLHIV in all regions except Western Africa, where initial breastfeeding was stable over time.

The median breastfeeding duration among HIV-negative mothers ranged from 18.3 months (95% CrI: 17.8–19.3) in Southern Africa to 24.6 months (95% CrI: 24.4–24.7) in Eastern Africa in 2010. In comparison with HIV-negative mothers, point estimates of median breastfeeding duration were shorter in MLHIV by 3%, 7%, 11%, and 15% in Western, Central, Eastern, and Southern Africa, respectively. Median breastfeeding duration decreased over time in all regions regardless of maternal HIV status.

### Sensitivity Analyses

**FIGURE 4.**
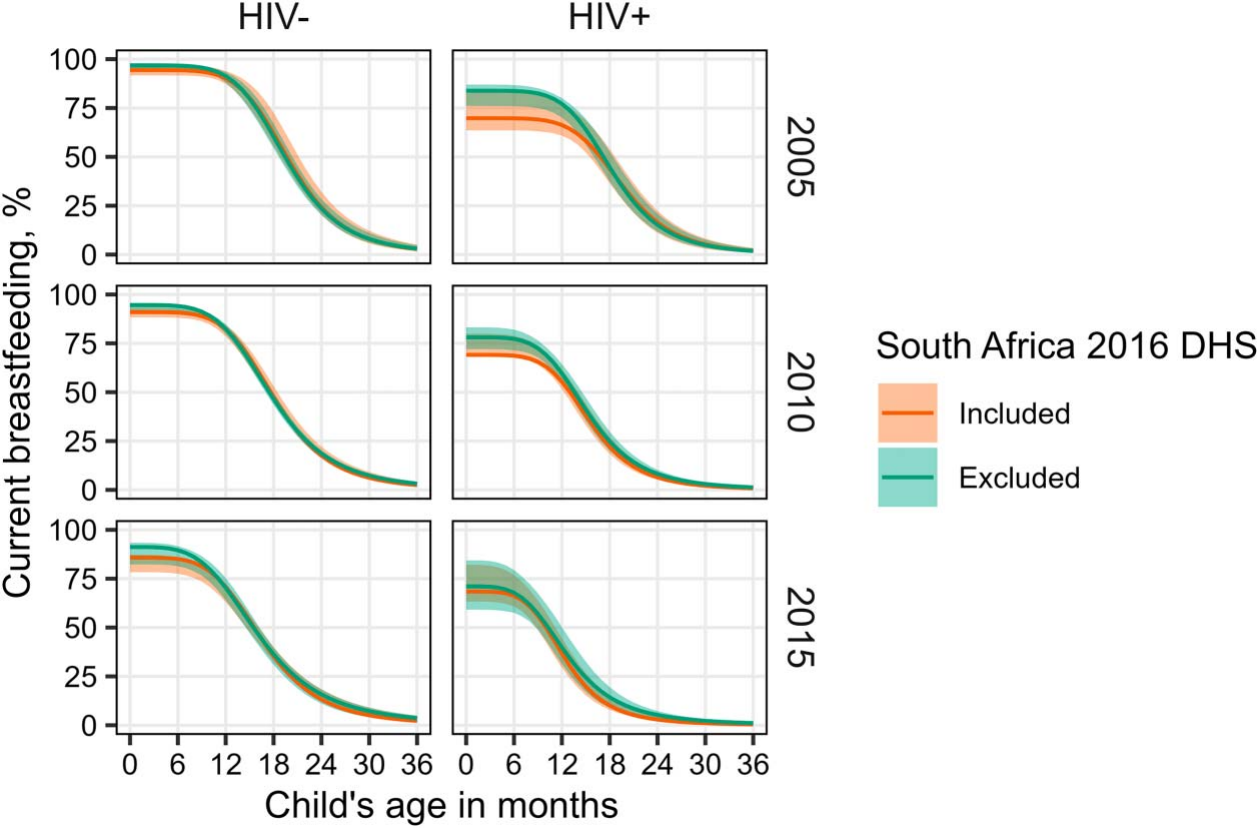
Sensitivity of Southern African breastfeeding estimates to inclusion of the 2016 DHS of South Africa. Regional trends are based on averages of country parameter estimates. Point estimates (solid curves) and 95% credible intervals (shaded areas) are shown.

We refitted the model to data from Southern Africa with 2016 DHS of South Africa excluded because the survey was performed later and found a short breastfeeding duration compared with other surveys in the region.^31^ Patterns of current breastfeeding in 2010 and 2015 were similar regardless of whether the 2016 DHS of South Africa was included, although differences among MLHIV in 2005 were more substantial (Fig. 4, Table 4, Supplemental Digital Content, http://links.lww.com/QAI/C133). Point estimates of initial breastfeeding among MLHIV mothers were higher if the 2016 DHS of South Africa was excluded (71% [95% CrI: 59–84] in 2015 versus 68% [95% CrI: 63–82] if included). The difference in initial breastfeeding among MLHIV was the largest in 2005 (84% [95% CrI: 76–87] when excluded versus 70% [95% CrI: 64–83] if included), although the 95% credible intervals overlapped at all time points evaluated. The estimated median breastfeeding duration among MLHIV was similar whether or not the 2016 DHS of South Africa was included, for example, 15.3 months (95% CrI: 14.3–16.6) in 2010 if excluded and 15.6 (95% CrI: 14.2–16.3) if included.

### Effect on Pediatric HIV Estimates

In comparison with breastfeeding durations for all mothers regardless of HIV status, basing breastfeeding durations in Spectrum on mothers living with HIV lowered estimates of pediatric HIV indicators in all 4 regions of sub-Saharan Africa in 2021. Estimates of new child infections were 9% lower and estimates of children living with HIV were 10% lower in sub-Saharan Africa overall. Proportionate differences in pediatric HIV estimates between breastfeeding assumptions were larger in Eastern and Southern Africa than in Western and Central Africa (Text 1, Supplemental Digital Content, http://links.lww.com/QAI/C133).

## DISCUSSION

We analyzed national household surveys to estimate proportions of mothers currently breastfeeding by maternal HIV status, over time, and between countries in sub-Saharan Africa. Our analyses were based on HIV serological testing performed within the surveys and include women who may not have known their HIV status before the survey. We found that, compared with HIV-negative mothers, mothers living with HIV were less likely to initiate breastfeeding and had shorter median duration of breastfeeding. Although these findings were consistent across regions of sub-Saharan Africa, the magnitude of differences in breastfeeding practices by maternal HIV status varied by region and was smallest in Western Africa and largest in Southern Africa. The median duration of breastfeeding decreased over time regardless of HIV status, and the proportion of mothers who breastfed initially did not increase in any region. These methods were first incorporated in annual UNAIDS-supported HIV estimates in 2020 and resulted in 9% lower estimates of new pediatric HIV infections compared with previous breastfeeding duration assumptions that did not account for maternal HIV status.^18^

Changes in breastfeeding practices over time and heterogeneity between sub-Saharan African countries have been reported previously.^32–34^ A geospatial analysis of African countries found substantial variation in exclusive breastfeeding in the first 6 months of life between and within countries and found that exclusive breastfeeding generally increased during 2000–2017.^32^ The finding that exclusive breastfeeding increased over time may seem contradictory given the decreases in initial breastfeeding we report. However, this could indicate that breastfeeding mothers' practices shifted from mixed feeding to exclusive breastfeeding. This is consistent with a study which found that exclusive breastfeeding in the first 6 months of life increased on average between 2000 and 2019 in sub-Saharan Africa despite stable or decreasing percentages of children receiving any breastfeeding at age 6 months or 1 year.^33^ These previous studies included most surveys included in our analysis. We are reassured that our restriction to surveys with HIV testing produced similar results.

Our findings that MLHIV were less likely to initiate breastfeeding and stopped breastfeeding sooner are consistent with cohort studies in several African countries. HIV-exposed uninfected infants were less likely be breastfed at age 9 months in comparison with HIV-unexposed uninfected infants in Kenya (72% vs. 98%)^35^ and South Africa (34.6% vs. 57.3%).^36^ Two other South African studies found that MLHIV were about twice as likely to not be breastfeeding at 14 weeks in comparison with HIV-negative mothers^37^ and that 93.1% of postnatal HIV-negative women initiated breastfeeding compared with 66.3% of MLHIV.^38^ A study in Ghana found that MLHIV were less likely to report any breastfeeding at 6, 9, or 12 months after birth compared with women who were HIV-negative or who did not know their HIV status.^39^ MLHIV groups in these studies knew their HIV-positive status, whereas our analyses include mothers who may have been unaware of their HIV status before the surveys.

We sought to describe patterns of breastfeeding among MLHIV for use in Spectrum for estimation of new HIV infections in children. Previous studies and reviews have examined the factors that influence breastfeeding decisions among MLHIV.^7,8^ Practices recommended by WHO for HIV-negative mothers have been consistent since 2001,^10,11^ but recommendations for MLHIV have varied, ranging from guidance in the early 2000s to end breastfeeding as early as possible to minimize mother-to-child transmission,^14^ by 6 months in 2007,^40^ by 12 months in 2010,^12^ or, as of 2016, to continue breastfeeding for 24 months or longer with support for adherence to antiretroviral therapy.^13^ Recommendations for the duration of exclusive breastfeeding and rapidity of weaning evolved as well. Countries may not have incorporated WHO recommendations into national guidelines immediately or in their entirety. Health care providers may not have always been aware of or comfortable with contemporary guidance, and mothers may have been confused by or distrustful of changing recommendations.^9,38,41–46^

Infant feeding and young child feeding decisions among MLHIV are subject to barriers and facilitators beyond the policy landscape. Fear of mother-to-child transmission is a frequently cited factor in breastfeeding decisions.^9,38,41,44,46–48^ HIV-related stigma may mitigate differences in breastfeeding practices by maternal HIV status. Mixed feeding in the first 6 months of life is not recommended by WHO but is common in sub-Saharan Africa.^46^ MLHIV may adopt these nonrecommended practices to avoid unintended HIV status disclosure.^38,44,45^

The national-level differences in breastfeeding practices we observed between HIV-positive and HIV-negative women may not be solely, directly determined by knowledge of HIV status. More generally, HIV status may be associated with factors such as socioeconomic, employment, and education status that are independently associated with breastfeeding decisions.^6,46^ For example, similar to differences in fertility by HIV status,^49^ breastfeeding practices may be systematically different in urban areas which also tend to have higher HIV prevalence. Although the purpose of this analysis was to derive appropriate assumptions for breastfeeding duration among MLHIV for national-level Spectrum estimates, because of this potential confounding, one should be cautious in extrapolating the national-level differences reported here to subnational populations. In future work, our mixture model regression framework could be extended to consider the potential role of confounders and mediating factors.

Key strengths of our analyses are the large numbers of surveys with similar methods and pooling of data within regions of sub-Saharan Africa. On their own, most surveys had too few MLHIV respondents to reliably estimate current breastfeeding practices at the age resolution needed by Spectrum, and most countries had too few surveys with HIV testing to estimate time trends reliably. Pooling data within regions allowed us to overcome these limitations. We were concerned that pooling this way risked misidentifying breastfeeding differences between countries in Southern Africa as a change over time because of the smaller number of surveys in the region and the relatively short breastfeeding duration in South Africa.^50^ We were reassured that a sensitivity analysis excluding the 2016 DHS of South Africa did not change regional estimates of breastfeeding patterns substantially.

Our analyses have several important limitations. We focus on estimates through 2015 because of the small number of eligible surveys with HIV testing in recent years. Few recent DHS include HIV testing, and several recent PHIA surveys did not collect current breastfeeding status, although the most recent PHIA2 surveys collect this and we plan to update our analyses when these survey data sets become available.^51^ Differences in breastfeeding practices by maternal HIV status may be smaller now because of convergence of breastfeeding recommendations for MLHIV and HIV-negative mothers.^13^ We estimated current practice of any breastfeeding that conform to Spectrum's input requirements^52^ while estimates that distinguish exclusive breastfeeding would be more applicable when monitoring progress toward global infant and young child nutrition targets.^53^ MLHIV who know their HIV-positive status may have different breastfeeding practices from those who do not; however, we were unable to account for the potential effects of HIV status awareness as DHS and AIS respondents are not asked about the results of any past HIV tests. Similarly, MLHIV receiving antiretroviral drugs (ARVs) for treatment or PMTCT know their HIV status and may receive more counseling on breastfeeding practices compared untreated MLHIV; however, we were unable to stratify breastfeeding estimates by ARV status because this was only ascertained in the 3 PHIA surveys included in our analysis. These knowledge gaps are important given that almost half of new pediatric HIV infections in 2022 were attributed to mothers who did not receive ART during pregnancy and breastfeeding.^1^ We analyzed breastfeeding duration based on time since last birth. Our estimates may be biased by missing breastfeeding data for older children of women who had multiple births within 36 months of each survey. Our analyses are restricted to countries in sub-Saharan Africa with nationally representative household surveys with HIV testing. Countries without surveys could use regional average breastfeeding patterns in Spectrum, although this will not account for between-country heterogeneity.

## CONCLUSIONS

Mothers living with HIV in sub-Saharan Africa are less likely to breastfeed, and those who do tend to stop breastfeeding sooner compared with HIV-negative mothers. Our estimated patterns of breastfeeding among MLHIV facilitate estimation of new HIV infections among children acquired during breastfeeding and have been used to prepare UNAIDS estimates since 2020. Consequently, estimates of mother-to-child HIV infections and children living with HIV are lower compared with previous assumptions that breastfeeding practices for MLHIV were the same as HIV-negative women. Our analyses highlight that mothers living with HIV should be supported in recommended breastfeeding practices and to adhere to antiretroviral drugs for HIV treatment and prevention of postnatal mother-to-child transmission. Health care providers should be supported to provide clear guidance about recommended infant and young child feeding practices.

## Supplementary Material

SUPPLEMENTARY MATERIAL
