# Supplementary material for: Differences in Breastfeeding Duration by Maternal HIV Status: A Pooled Analysis of Nationally Representative Surveys in Sub-Saharan Africa
Source: J Acquir Immune Defic Syndr. 2024 Jan 4;95(1 Suppl):e81–8. doi: 10.1097/QAI.0000000000003317 (PMC10769179; doi:10.1097/QAI.0000000000003317)
Supplement: SUPPLEMENTARY MATERIAL [file qai-95-e81-s001.docx]

Text S1: Potential effects of breastfeeding duration on pediatric HIV estimates in Sub-Saharan Africa

Supplement to “Differences in breastfeeding duration by maternal HIV status: a pooled analysis of nationally representative surveys in sub-Saharan Africa”

Robert Glaubius, John Stover, Leigh F. Johnson, Severin G. Mahiane, Mary I. Mahy, Jeffrey W. Eaton

# Methods

We used Spectrum to evaluate the effects of different breastfeeding duration assumptions on incident and prevalent HIV infections in children. We used Spectrum models for 44 countries in sub-Saharan Africa (Table 1) that were submitted to UNAIDS for the 2022 HIV estimates round. A complete overview of Spectrum demographic and HIV epidemic calculations are available elsewhere[^1-3^](#_ENREF_1). Pertinent to the present analysis, projections typically ran from 1970 to 2025 or later and took as input the proportion of mothers who breastfed by year and child’s age (0-1, 2-3, …, 34-35 months). Spectrum accounts for breastfeeding duration and use of antiretroviral drugs among mothers living with HIV when calculating postnatal mother-to-child HIV transmission.

We compared pediatric HIV estimates when using breastfeeding durations among a) mothers living with HIV or b) all mothers regardless of HIV status. In the latter, we averaged breastfeeding durations among mothers living with or without HIV weighted by national HIV prevalence. We used Spectrum estimates of HIV prevalence in pregnant women over time in each country as a proxy for HIV prevalence among mothers with children aged <36 months. We derived breastfeeding inputs for the entire projection period by calculating the breastfeeding survival model by year within the period spanned by surveys in each region, then assumed breastfeeding patterns were constant before the earliest and after the latest survey in the region. Breastfeeding duration inputs were based on the best-fitting parameter point estimates. Thirteen of 44 countries did not have household survey data for breastfeeding duration estimation (Table 1). We used regional average breastfeeding duration estimates in these countries.

# Results

Breastfeeding durations for all mothers resulted in an estimated 141,800 new child HIV infections and 1,628,700 children living with HIV in sub-Saharan Africa in 2021 (Table 2). In comparison, breastfeeding durations specific to mothers living with HIV lowered estimated new child infections by 9.1% (128,900) and children living with HIV by 10.1% (1,464,300). Estimates decreased in all regions when breastfeeding durations for mothers living with HIV were used: new child infection estimates were lower by 2.7% in Western Africa, 6.3% in Central Africa, 9.5% in Eastern Africa, and 26.1% in Southern Africa, while prevalent infection estimates were lower in these regions by 4.1%, 8.9%, 9.5%, and 16.6%, respectively. Absolute differences in estimated new child infections between breastfeeding duration assumptions were largest in 1995-2007 across regions, while absolute differences in estimated children living with HIV were largest in 2006-2010 (Figure 1).

**Table 1. Countries included in the analysis of pediatric HIV indicators.**

| Region | Countries |
| --- | --- |
| Central Africa | Angola, Cameroon, Central African Republic*, Chad, Congo*, Democratic Republic of Congo*, Equatorial Guinea*, Gabon, São Tomé and Príncipe |
| Eastern Africa | Burundi, Comoros*, Eritrea*, Ethiopia, Kenya, Madagascar*, Malawi, Mozambique, Rwanda, South Sudan*, Uganda, United Republic of Tanzania, Zambia, Zimbabwe |
| Southern Africa | Botswana*, Eswatini, Lesotho, Namibia, South Africa |
| Western Africa | Benin*, Burkina Faso, Cabo Verde*, Côte d’Ivoire, Gambia, Ghana, Guinea, Guinea-Bissau*, Liberia, Mali, Mauritania*, Niger, Nigeria*, Senegal, Sierra Leone, Togo |

* Regional average breastfeeding duration estimates used.

**Table 2. Pediatric HIV indicator estimates for 2021 under different breastfeeding assumptions.**

|  | Breastfeeding duration based on all mothers | Breastfeeding duration based on HIV+ mothers | % decrease  (HIV+ vs. all) |
| --- | --- | --- | --- |
| New HIV infections in children |  |  |  |
| Central Africa | 20,600 | 19,300 | 6.3 |
| Eastern Africa | 69,200 | 62,600 | 9.5 |
| Southern Africa | 15,300 | 11,300 | 26.1 |
| Western Africa | 36,700 | 35,700 | 2.7 |
| Total | 141,800 | 128,900 | 9.1 |
|  |  |  |  |
| Children living with HIV |  |  |  |
| Central Africa | 183,100 | 166,800 | 8.9 |
| Eastern Africa | 779,700 | 705,600 | 9.5 |
| Southern Africa | 373,100 | 311,100 | 16.6 |
| Western Africa | 292,800 | 280,800 | 4.1 |
| Total | 1,628,700 | 1,464,300 | 10.1 |


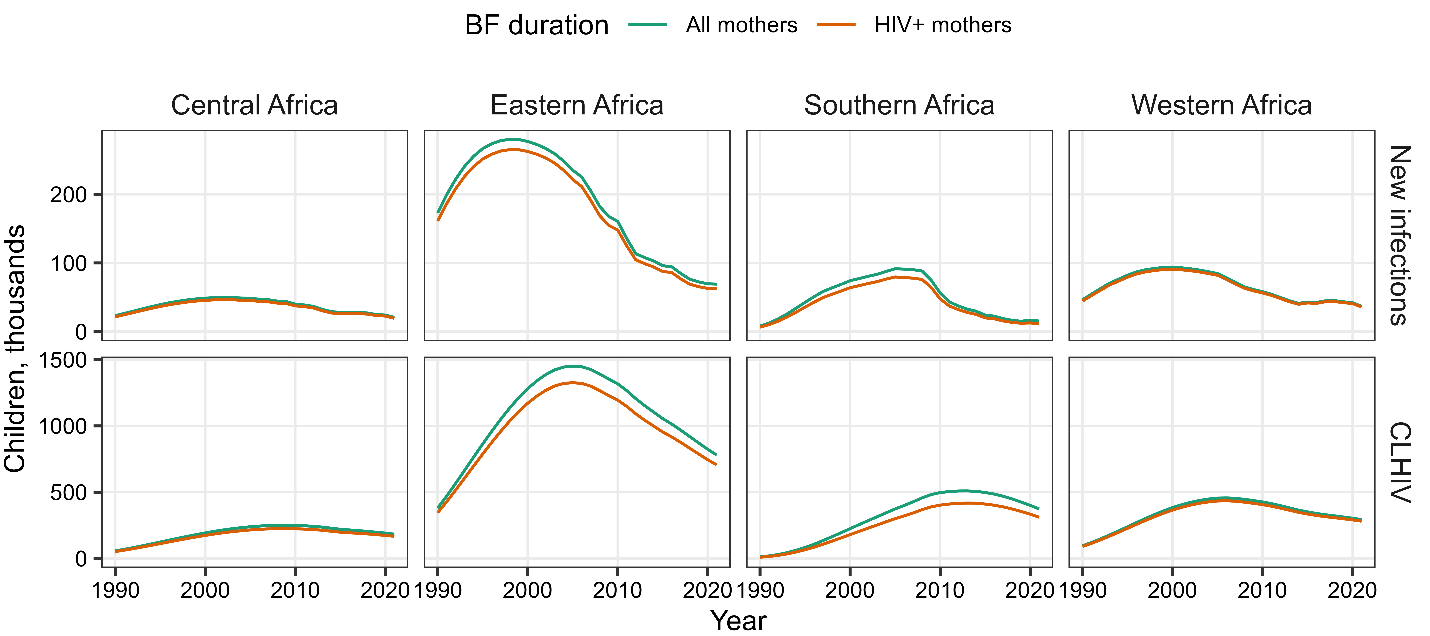


**Figure 1. Trends in new child infections (top) and children living with HIV (bottom).** Estimates were produced in Spectrum when basing breastfeeding duration inputs on all mothers (green) or mothers living with HIV (orange) in household surveys. CLHIV, children living with HIV.

# Discussion

Before the 2020 round of UNAIDS HIV estimates, new HIV infections in children in sub-Saharan Africa were often estimated using breastfeeding durations measured in household surveys among all mothers regardless of HIV status[^4^](#_ENREF_4). This approach likely led to overestimation of pediatric HIV burden, especially in Eastern and Southern Africa where HIV prevalence is higher and differences in breastfeeding practices by maternal HIV status are larger. While shorter breastfeeding durations reduced estimates of pediatric HIV burden in Spectrum, we do not recommend discouraging mothers living with HIV from breastfeeding. Breastfeeding improves child health and survival, but aside from differences in HIV acquisition risk Spectrum does not account for differences in child morbidity and mortality when varying breastfeeding assumptions. Rather, the shorter breastfeeding durations we estimated are intended to improve pediatric HIV estimates via more accurate representation of breastfeeding practices among mothers living with HIV and improved consistency with data that inform child mortality in Spectrum.

While we used Spectrum models submitted to UNAIDS for the 2022 HIV estimates round, the indicator estimates here differ somewhat from UNAIDS estimates from that round. Some national HIV estimates teams may have chosen not to use model-based breastfeeding estimates in their Spectrum files. We used DHS region definitions for countries with household surveys, which differ from UNAIDS definitions for Angola and Burundi. Finally, we used a newer Spectrum version (6.29) than was used in the 2022 HIV estimates round. These software changes may affect precise indicator values but are not expected to affect proportionate effects of varying breastfeeding assumptions meaningfully.

REFERENCES

1. Stover J, Glaubius R, Mofenson L, et al. Updates to the Spectrum/AIM model for estimating key HIV indicators at national and subnational levels. *AIDS.* 2019;33(Suppl 3):S227-S234.

2. Stover J, Glaubius R, Kassanjee R, Dugdale CM. Updates to the Spectrum/AIM model for the UNAIDS 2020 HIV estimates. *J Int AIDS Soc.* 2021;24(S5):e25778.

3. Avenir Health. Spectrum Manual: Spectrum System of Policy Models. <http://avenirhealth.org/Download/Spectrum/Manuals/SpectrumManualE.pdf>. Accessed Feb. 16, 2017.

4. Stover J, Brown T, Marston M. Updates to the Spectrum/Estimation and Projection Package (EPP) model to estimate HIV trends for adults and children. *Sex Transm Infect.* 2012;88(Suppl 2):i11-i16.

**Table S1.** **Regional parameter estimates.** Point estimates (95% credible intervals) are shown.

| Parameter | Central Africa | Eastern Africa | Southern Africa | Western Africa |
| --- | --- | --- | --- | --- |
| Change over time in initial BF ($\theta_{r}^{'}$) | -0.18  (-0.26 to -0.13) | -0.12  (-0.16 to -0.10) | -0.10  (-0.23 to -0.06) | -0.08  (-0.11 to -0.05) |
| Change over time in median BF duration ($\mu_{r}^{'}$) | -0.01  (-0.01 to 0.00) | -0.01  (-0.01 to 0.00) | -0.02  (-0.03 to -0.01) | 0.00  (-0.01 to 0.00) |
| Change over time in shape parameter ($\sigma_{r}^{'}$) | -0.01  (-0.01 to 0.01) | 0.01  (0.00 to 0.01) | -0.02  (-0.06 to -0.01) | 0.01  (0.00 to 0.02) |
| HIV effect on initial BF % ($\rho_{r}$) | -2.05  (-2.63 to -1.55) | -1.85  (-2.06 to -1.58) | -1.51  (-1.62 to -0.90) | -1.22  (-1.43 to -0.06) |
| Change over time in HIV effect on initial BF ($\rho_{r}^{'}$) | 0.11  (0.01 to 0.24) | 0.06  (0.02 to 0.12) | 0.10  (0.06 to 0.21) | 0.10  (-0.03 to 0.27) |
| HIV effect on median BF duration ($\lambda_{r}$) | -0.07  (-0.14 to 0.01) | -0.11  (-0.12 to -0.09) | -0.16  (-0.26 to -0.13) | -0.03  (-0.08 to 0.01) |
| Change over time in HIV effect on median BF duration ($\lambda_{r}^{'}$) | 0.01  (-0.01 to 0.02) | 0.00  (-0.01 to 0.00) | -0.03  (-0.04 to -0.01) | 0.00  (-0.01 to 0.01) |

BF, breastfeeding.

**Table S2. Country parameter estimates.** Point estimates (95% credible intervals) are shown.

| Country | Initial BF % among HIV-negative mothers  ($\boldsymbol{\theta}_{\boldsymbol{k}}$) | Median BF duration, months among HIV-negative mothers ($\mathbf{exp}\left( \boldsymbol{\mu}_{\boldsymbol{k}} \right)$) | Shape parameter ($\mathbf{exp}\left( \boldsymbol{\sigma}_{\boldsymbol{k}} \right)$) |
| --- | --- | --- | --- |
| **Central Africa** |  |  |  |
| Angola | 96.3 (95.1-97.6) | 21.7 (21.1-22.2) | 8.1 (6.5-8.2) |
| Cameroon | 98.2 (97.3-98.9) | 17.7 (17.3-17.9) | 5.4 (5.0-5.7) |
| Chad | 98.0 (97.7-99.0) | 24.0 (23.1-24.4) | 6.1 (4.8-6.0) |
| Democratic Republic of the Congo | 99.4 (99.0-99.6) | 24.4 (23.7-24.5) | 4.7 (4.5-5.2) |
| Gabon | 94.4 (89.5-95.3) | 12.3 (11.8-13.5) | 3.9 (3.5-4.7) |
| São Tomé and Príncipe | 99.2 (96.4-99.5) | 18.3 (17.5-18.8) | 7.4 (6.4-8.8) |
|  |  |  |  |
| **Eastern Africa** |  |  |  |
| Burundi | 99.1 (98.6-99.3) | 30.4 (30.2-31.8) | 4.4 (3.9-4.8) |
| Ethiopia | 98.0 (97.6-98.5) | 32.7 (31.7-33.0) | 3.6 (3.5-4.0) |
| Kenya | 98.3 (98.0-99.1) | 22.4 (22.0-22.8) | 4.6 (4.2-4.8) |
| Malawi | 98.0 (97.7-98.5) | 25.2 (25.1-25.6) | 8.3 (8.0-9.0) |
| Mozambique | 98.6 (97.9-99.0) | 22.9 (22.3-23.3) | 7.1 (6.6-8.1) |
| Rwanda | 99.4 (99.0-99.7) | 34.7 (33.9-36.2) | 3.7 (3.2-3.9) |
| Tanzania | 99.2 (98.7-99.4) | 21.6 (21.3-21.8) | 6.5 (6.2-6.9) |
| Uganda | 98.6 (97.9-99.0) | 20.6 (20.0-20.8) | 5.1 (4.8-5.5) |
| Zambia | 98.9 (98.6-99.1) | 21.1 (21.0-21.4) | 7.9 (7.4-8.2) |
| Zimbabwe | 98.9 (98.5-99.1) | 19.1 (18.9-19.3) | 8.4 (8.1-9.0) |
|  |  |  |  |
| **Southern Africa** |  |  |  |
| Eswatini | 90.4 (85.8-93.6) | 18.2 (16.9-18.7) | 6.7 (4.5-6.6) |
| Lesotho | 96.5 (94.0-96.9) | 19.2 (19.0-20.4) | 4.5 (4.1-5.2) |
| Namibia | 95.1 (92.0-97.6) | 16.7 (15.5-18.0) | 4.4 (3.8-5.6) |
| South Africa | 81.9 (74.4-90.2) | 19.4 (17.7-23.0) | 6.0 (3.8-8.0) |
|  |  |  |  |
| **Western Africa** |  |  |  |
| Burkina Faso | 98.9 (98.4-99.3) | 25.9 (25.6-26.2) | 8.1 (7.2-8.4) |
| Côte d'Ivoire | 96.4 (95.5-97.9) | 20.1 (19.7-20.8) | 6.6 (5.8-7.3) |
| Gambia | 98.8 (98.1-99.4) | 21.6 (21.2-22.1) | 9.1 (8.0-10.3) |
| Ghana | 99.0 (99.0-99.8) | 22.7 (22.1-22.9) | 7.7 (6.6-7.8) |
| Guinea | 97.3 (96.8-98.0) | 24.1 (23.7-24.5) | 6.1 (5.7-6.5) |
| Liberia | 97.6 (97.1-98.9) | 20.3 (20.0-20.9) | 5.5 (4.9-5.7) |
| Mali | 97.6 (96.6-98.2) | 23.4 (23.2-24.0) | 5.7 (5.3-6.2) |
| Niger | 98.4 (98.0-99.1) | 21.9 (21.8-22.4) | 6.8 (6.6-7.6) |
| Senegal | 98.5 (98.2-99.0) | 21.4 (21.2-21.7) | 7.9 (7.3-8.2) |
| Sierra Leone | 97.0 (96.3-97.9) | 21.0 (20.6-21.3) | 5.5 (5.0-5.8) |
| Togo | 97.9 (97.0-98.9) | 24.5 (23.9-25.1) | 6.7 (5.9-7.6) |

BF, breastfeeding.

**Table S3. Regional average estimates of initial breastfeeding and median breastfeeding duration.** Point estimates (95% credible intervals) are shown. Regional estimates are based on the average of country parameter estimates in Table S2.

|  |  | HIV-negative mothers | | HIV-positive mothers | |
| --- | --- | --- | --- | --- | --- |
| **Region** | **Year** | **Initial BF %** | **Median BF duration, months** | **Initial BF %** | **Median BF duration, months** |
| Central Africa | 2005 | 99.0 (98.1-99.4) | 19.8 (19.3-20.3) | 88.1 (73.7-93.6) | 17.9 (16.6-21.0) |
|  | 2010 | 97.6 (96.4-98.0) | 19.2 (18.9-19.5) | 83.8 (73.2-88.0) | 17.9 (16.6-19.5) |
|  | 2015 | 94.1 (91.5-94.6) | 18.7 (18.3-18.9) | 78.3 (66.1-84.0) | 17.9 (15.9-19.3) |
|  |  |  |  |  |  |
| Eastern Africa | 2005 | 99.3 (99.1-99.5) | 25.3 (25.1-25.6) | 94.2 (92.4-96.0) | 22.6 (22.3-23.7) |
|  | 2010 | 98.7 (98.5-98.9) | 24.6 (24.4-24.7) | 92.2 (91.0-93.9) | 22.0 (21.7-22.5) |
|  | 2015 | 97.6 (97.3-97.8) | 23.8 (23.7-24.0) | 89.8 (88.1-91.8) | 21.3 (20.8-21.6) |
|  |  |  |  |  |  |
| Southern Africa | 2005 | 94.4 (91.7-97.5) | 20.1 (19.1-21.5) | 69.8 (63.6-83.4) | 19.7 (17.8-21.0) |
|  | 2010 | 91.0 (88.3-93.2) | 18.3 (17.8-19.3) | 69.1 (68.0-79.9) | 15.6 (14.2-16.3) |
|  | 2015 | 85.8 (78.4-87.2) | 16.7 (16.1-17.8) | 68.4 (63.3-82.1) | 12.4 (10.7-13.5) |
|  |  |  |  |  |  |
| Western Africa | 2005 | 98.6 (98.4-99.0) | 22.8 (22.7-23.1) | 92.8 (88.1-98.4) | 22.4 (21.0-24.2) |
|  | 2010 | 98.0 (97.9-98.4) | 22.4 (22.3-22.6) | 93.4 (92.7-98.0) | 21.7 (20.6-22.7) |
|  | 2015 | 97.0 (96.8-97.6) | 22.0 (21.8-22.1) | 93.9 (92.7-98.7) | 21.0 (19.4-22.1) |

BF, breastfeeding.

**Table S4. Sensitivity of estimates of initial breastfeeding and median breastfeeding duration in Southern Africa to South Africa’s 2016 DHS**. Point estimates (95% credible intervals) are shown. Regional estimates are based on the average of country parameter estimates.

|  |  | HIV-negative mothers | | HIV-positive mothers | |
| --- | --- | --- | --- | --- | --- |
| **Include South Africa’s 2016 DHS?** | **Year** | **Initial BF %** | **Median BF duration, months** | **Initial BF %** | **Median BF duration, months** |
| Yes | 2005 | 94.4 (91.7-97.5) | 20.1 (19.1-21.5) | 69.8 (63.6-83.4) | 19.7 (17.8-21.0) |
|  | 2010 | 91.0 (88.3-93.2) | 18.3 (17.8-19.3) | 69.1 (68.0-79.9) | 15.6 (14.2-16.3) |
|  | 2015 | 85.8 (78.4-87.2) | 16.7 (16.1-17.8) | 68.4 (63.3-82.1) | 12.4 (10.7-13.5) |
|  |  |  |  |  |  |
| No | 2005 | 96.7 (94.9-98.0) | 19.7 (18.9-20.6) | 83.8 (76.1-87.0) | 18.4 (17.3-20.1) |
|  | 2010 | 94.6 (92.0-95.5) | 17.9 (17.4-18.5) | 78.1 (72.0-83.2) | 15.3 (14.3-16.6) |
|  | 2015 | 91.2 (82.3-93.4) | 16.2 (15.4-17.3) | 71.1 (59.2-84.4) | 12.8 (11.0-14.6) |
